# Criminal Abortion

**Published:** 1869-10

**Authors:** 


### Criminal Abortion.

Dr. Ira D. Hopkins, of Utica, offered to the Oneida County Medical Society, at its last meeting, Oct. 11th, the following preamble and resolutions on the subject of criminal abortion, which were adopted unanimously:

Whereas, We, the members of the Oneida Medical Society, are cognizant of the fact that the crime of procured abortion is existing to an alarming extent and fearfully growing in all classes of society; and, whereas, we know well that abortion is the sacrifice of human life, and that the period of time when procured does not diminish the atrociousness of the crime; and, whereas, it is apparent by the homicides which are continually occurring in our city and county, that we, as respectable, conscientious medical men of the said city and county, are called to let the public understand our moorings in respect to this, one of the most gigantic evils of the day; therefore, be it

*Resolved*, That the members of the Oneida County Medical Society are willing and desirous to co-operate with all organizations in using every means to diffuse a knowledge of the turpitude of procured abortions, which is so generally regarded by all classes as without a shadow of guilt, and resorted to by very many who would tremble at the thought of taking human life.

*Resolved*, That we, the members of this society, acquiesce in the opinion of the most eminent, profound and learned of our profession, that the fœtus is a living being from the earliest period of gestation. the destruction of whose life is as heinous a crime as infanticide, and that those who produce its death are murderers.

*Resolved*, That it is the duty of every member of this society, not only positively and firmly to decline, when applied to for this purpose, but to put forth all his influence to hinder its completion, by showing its criminality, and disabusing, as far as possible, the false belief, which is so prevalent respecting the life of the fœtus.

*Resolved*, That this society will not admit nor retain in its fellowship, any physician or surgeon who shall be known to practice this iniquitous business, nor even those who treat this subject with so much indifference that they sanction rather than discountenance the crime.

